# Learning in the Single-Cell Organism *Physarum polycephalum*: Effect of Propofol

**DOI:** 10.3390/ijms24076287

**Published:** 2023-03-27

**Authors:** Stefan Kippenberger, Gordon Pipa, Katja Steinhorst, Nadja Zöller, Johannes Kleemann, Deniz Özistanbullu, Roland Kaufmann, Bertram Scheller

**Affiliations:** 1 Department of Dermatology, Venereology and Allergy, Johann Wolfgang Goethe University, 60590 Frankfurt, Germany; 2 Institute of Cognitive Science, University Osnabrück, 49074 Osnabrück, Germany; 3 Department of Anesthesia, St. Josefs-Hospital, 65189 Wiesbaden, Germany

**Keywords:** propofol, anesthetics, memory, learning, single-celled organism, *Physarum polycephalum*

## Abstract

Propofol belongs to a class of molecules that are known to block learning and memory in mammals, including rodents and humans. Interestingly, learning and memory are not tied to the presence of a nervous system. There are several lines of evidence indicating that single-celled organisms also have the capacity for learning and memory which may be considered as basal intelligence. Here, we introduce a new experimental model for testing the learning ability of *Physarum polycephalum*, a model organism frequently used to study single-celled “intelligence”. In this study, the impact of propofol on *Physarum*’s “intelligence” was tested. The model consists of a labyrinth of subsequent bifurcations in which food (oat flakes soaked with coconut oil-derived medium chain triglycerides [MCT] and soybean oil-derived long chain triglycerides [LCT]) or propofol in MCT/LCT) is placed in one of each Y-branch. In this setting, it was tested whether *Physarum* memorized the rewarding branch. We saw that *Physarum* was a quick learner when capturing the first bifurcations of the maze; thereafter, the effect decreased, perhaps due to reaching a state of satiety. In contrast, when oat flakes were soaked with propofol, *Physarum*’s preference for oat flakes declined significantly. Several possible actions, including the blocking of gamma-aminobutyric acid (GABA) receptor signaling, are suggested to account for this behavior, many of which can be tested in our new model.

## 1. Introduction

The history of multicellular organisms on our planet is relatively young and dates back about 800 million years, whereas that of single-celled organisms starts about 3.8 billion years ago. For a long time, the behavioral spectrum of single-celled organisms was considered as rather limited, allowing only stereotypic reactions to a stimulus. Recently, new observations have suggested that this view is no longer valid and that single cells—with no correlation to a brain-like structure—are capable of complex behavior. There are some impressive examples: bacteria were considered to possess social intelligence by, e.g., quorum sensing and decision making concerning colony structure and thereby generating an inheritable colonial memory [1,2]. *Paramecium caudatum*, a single-celled aquatic organism, can be trained to discriminate between dark and light by electric shocks [3]. *Tetrahymena*, a relative of *Paramecium*, memorizes its environment. After its release from a minute water droplet into a larger area, it recapitulates the circular swimming trajectories learnt in captivity [4]. The vast majority of experiments in this context have been performed with the unicellular slime mold *Physarum polycephalum*. When placed in a labyrinth with two exits where *Physarum’s* preferred food, oat flakes, was deposited, the cell retracted from all detours and dead-end branches leaving only a linear connection of the shortest distance [5]. In a related experiment, *Physarium* solved the complex traveling-salesman problem by linking points, represented by oat flakes, with the shortest distances [6,7]. *Physarum* as a habitant of dark and wet environments does not like dryness. When irritated by cool and dry air, it reduces its running speed. After a few irritations, the cell anticipates the next irritation and reduces the running speed without environmental changes in temperature and humidity [8]. Particularly impressive are experiments showing that *Physarum* can learn to ignore repelling conditions when they are associated with a reward in the form of a source of nutrients. In this context, *Physarum* was conditioned to colder temperatures, which it normally avoids, when food was offered there [9,10]. Similarly, the effect of chemical repellents could be reversed when contact with them was rewarded with food [11]. Of note, this work shows that *Physarum* can transfer the learned behavior to other individuals by cell fusion. This observation indicates a material correlate for learning. To date, there is no convincing theory about the nature of such a material correlate.

In this paper, we pursue the idea that anesthetics used in humans may also have an impact on learning in single-celled organisms. Postoperative delirium is a common condition after the administration of anesthetics which is characterized by altered consciousness, disorientation, impaired memory, perceptual disturbance, altered psychomotor activity, and altered sleep–wake cycles [12]. Our results, although preliminary, indicate that propofol—which is known as the “milk of amnesia” [13]—also depresses learning and memory in *Physarum*.

## 2. Results

### 2.1. Learning in the 5-Level Y-Maze

**Figure 1:**
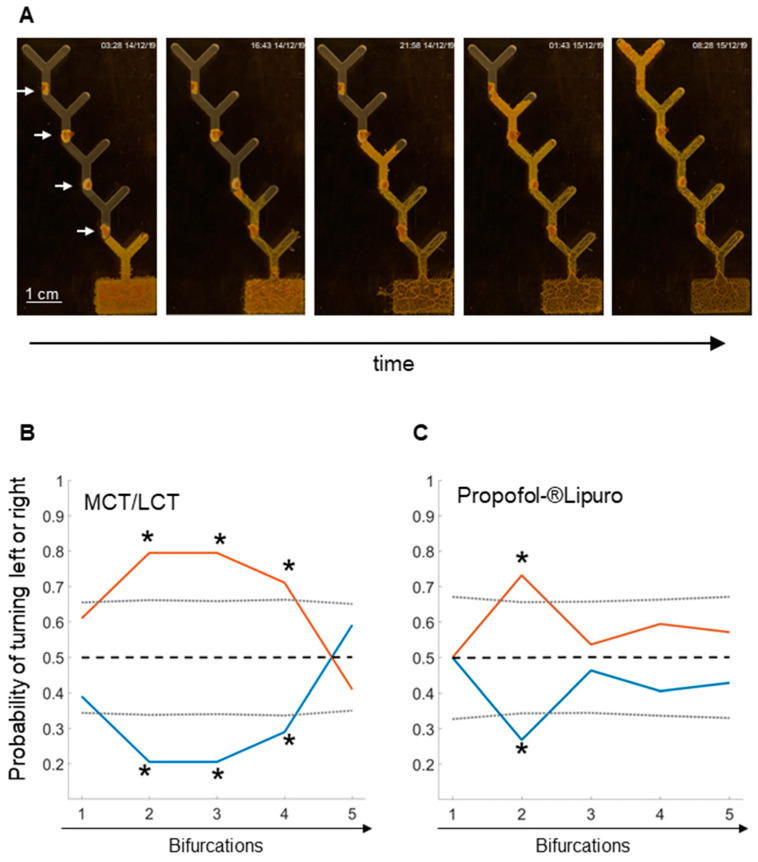
**The 5-level Y-maze:** (**A**) Exemplary course of an experiment with *Physarum* in a 5-level Y-maze. Starting with a rectangular chamber, *Physarum* spreads out along successive bifurcations. At each of the first 4 bifurcations, *Physarum*’s favorite food, an oat flake, is placed at the tip of the left arm (see white arrows). The oat flakes are either soaked with MCT/LCT or Propofol-^®^Lipuro. The fifth bifurcation is devoid of an oat flake. (**B**) Probability representation of *Physarum*´s directional decisions at each bifurcation in the 5-level Y-maze with oat flakes soaked with MCT/LCT or (**C**) Propofol-^®^Lipuro. The red lines represent the decision to the left (oat) and the blue lines to the right (no oat). The dashed black lines indicate the mean, and the grey dotted lines indicate the confidence intervals of two times the standard deviation. Values greater or less than this limit indicate significance (* = *p* < 0.05). The graphs represent 50 experiments for each condition (MCT/LCT or Propofol-^®^Lipuro).

The medium within the maze consisted of 1.25% agar, providing a humid substrate to avoid desiccation, but was devoid of nutrients. After inoculation in the rectangular starting chamber, it took 1 to 2 days before *Physarum* started spreading into the maze. At the tip of the left branch of the first Y, *Physarum* was rewarded with an oat flake to satisfy the initial hunger. This procedure was repeated for the next three decisions. At the last bifurcation, no oat flake was given in order to test *Physarum*’s memory retention for the rewarding branch. The whole procedure from the start to the end of the maze took between 3 and 6 days. Figure 1A displays an exemplary run. In order to test the effect of propofol, we started with the control where the oat flakes were soaked with MCT/LCT, the solvent of Propofol-^®^Lipuro. Figure 1B shows the summary of 50 runs as a probability for the preferred directions *Physarum* took at each bifurcation.

Already at the second bifurcation, *Physarum* showed a distinct preference for the left branch (probability = 0.79); the probability for turning to the right branch was 0.21. The clear preference for the left remained for bifurcation 3 (probability = 0.79) and 4 (probability = 0.71). The calculated probabilities at bifurcation 2, 3, and 4 were significant with *p*-values < 0.05. At the final bifurcation with no rewarding oat flake, the probability for turning to the left was 0.41 and not significant. In the following experiment, we investigated the effect of propofol in the aforementioned model by offering *Physarum* oat flakes soaked with Propofol-^®^Lipuro. The results of 50 runs are shown in Figure 1C. The decision for the left fork at the second bifurcation was lower (probability = 0.73) than in the control (probability = 0.79); the probability for the nonrewarding option (right) was 0.27. However, the probability for turning left at the second bifurcation was significant. In the further course, at bifurcation 3, the probability for the rewarding decision (left) decreased (reward 0.54 vs. nonreward 0.46) showing no statistical significance. At bifurcation 4, the probability for turning to the rewarding side was 0.59 and the probability of turning to the nonrewarding side was 0.41, both being not significant. At bifurcation 5, the probability for turning to the rewarding side was 0.57 and 0.43 for the non-rewarding side, respectively. In summary, under control conditions with MCT/LCT, the probabilities of choosing the rewarding side were significantly higher than in the experiments with propofol.

### 2.2. Propofol Repels Physarum in the 1-Level Y-Maze

**Figure 2:**
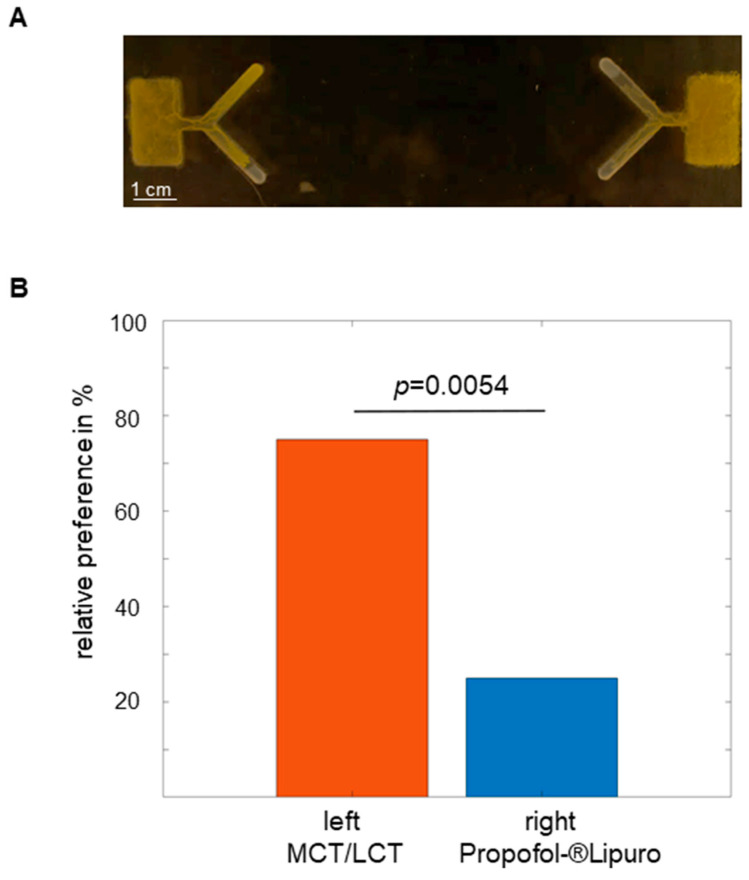
**The 1-level Y-maze:** (**A**) Exemplary experiment with *Physarum* in a dual 1-level Y-maze. Starting with a rectangular chamber, *Physarum* spreads out into the arms of the Y. A depot consisting of 3 µL of MCT/LCT was placed at the tip of the left arm; correspondingly, a depot consisting of 3 µL of Propofol-^®^Lipuro was placed at the tip of the right arm. (**B**) Percentage representation of *Physarum*´s directional decisions (total 30 experiments).

In this section, it was tested whether Propofol-^®^Lipuro or MCT/LCT had an effect on *Physarum*´s preference for one direction. For this purpose, two opposite rectangles with one extending Y each were milled in acrylic glass and filled with 1.25% agar as described above. The tips of each branch were filled with 3 µL of Propofol-^®^Lipuro (right) and 3 µL of MCT/LCT (left) using a pipette. The starting chamber was inoculated with Physarum and placed into the acrylic box for observation. No food in the form of oat flakes was supplied. Over the following 2–3 days, the *Physarum* spread into the Y-maze. An exemplary image is shown in Figure 2A.

Quantitative decisions for Propofol-^®^Lipuro or MCT/LCT from 30 experiments are shown in Figure 2B. It was found that in 75% of all experiments, *Physarum* turned to the MCT/LCT arm and 25% of all experiments to the propofol arm. This difference was statistically significant (*p* = 0.0054). These results indicate a distinct aversion to propofol.

### 2.3. GABA Receptor Transcripts in Physarum

The transcriptome *Physarum* cultivated under regular conditions is available under http://www.physarum-blast.ovgu.de (accessed on 17 November 2022) [14]. The deposited transcriptome was queried for similarities in the protein sequence to the human and rat GABA receptor. Table 1 shows the first three hits of the query. High-score bits and the low E-values indicate the presence of GABA receptor transcripts in *Physarum*.

**Figure 3:**
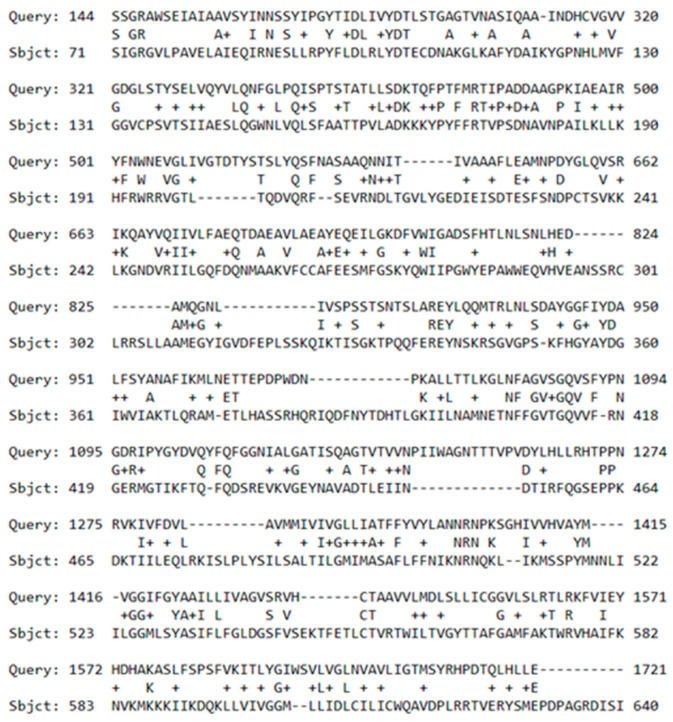
**Transcript alignment to GABA receptor.** Alignment section of the first hit between *Physarum* transcript 03383 and the protein sequence O88871 coding for the GABA type B receptor subunit 2 in Rattus norwegicu.

Figure 3 shows a section of the alignment between *Physarum* transcript 03383 and the GABA type B receptor subunit 2 in *Rattus norwegicus*. This is a first indication of a GABA receptor homologue present in Physarum.

## 3. Discussion

The work herein presented introduces a new model for the detection of learning and memory in *Physarum* using a five-level Y-maze. The experiment is based on the assumption that repeated rewards with food at one point in a decision tree influences *Physarum*’s decision for future feedings. When the experiment was designed, we thought that repeated rewards on the left branch in a binary decision situation would steepen the learning curve, amplifying the preference for the left. Indeed, after the first food exposition (oat flake soaked with MCT/LCT) in the left branch, *Physarum* showed a clear preference for the left at the second bifurcation. Of note, this effect continued for the subsequent bifurcations 3 and 4, but was not statistically significant anymore at bifurcation 5. Future experiments with a shorter maze and different combinations of reward oats will help clarify this question. In general, the decision finding of *Physarum* can be described by the concept of predictive processing in which an organism optimizes predictions to minimize the sensory prediction error [15,16].

In the following experiment, we pursued the hypothesis that propofol may have an impact on *Physarum*’s memory and learning capacity in the aforementioned model by offering *Physarum* oat flakes soaked with 1% Propofol-^®^Lipuro, which is a much higher concentration than that used for sedation in humans, ranging from 1 to 4 mg/kg/hr. However, this concentration did not stop *Physarum*´s motility. The experiment showed that the overall preference for the food-rewarding branch was much less pronounced than in the control experiment with MCT/LCT. In particular, no preference for the propofol-soaked oat flake was measured at bifurcation 3.

The mechanism by which anesthetics interfere with consciousness and memory in humans is still elusive, which is rather surprising considering that these agents are given to millions of patients each year. Propofol alone, for example, puts 30–50 million patients per year to sleep in the USA [13]. There is now a consensus that propofol binds to the pentameric GABA_A_ receptor; however, it is still a conundrum how this event triggers the transition from consciousness to unconsciousness [13]. Common to all anesthetics, despite their chemical dissimilarities, is that anesthetic potency correlates over many orders of magnitude with one factor: the solubility in a lipid medium coined by the famous Meyer–Overton rule [17]. Therefore, it seemed natural to suspect changes in the physicochemical properties of neural lipid membranes being responsible for the anesthetic effect. However, anesthetics also have an effect on organisms without a nervous system, e.g., the ciliated protozoan *Tetrahymena pyriformis* becomes immobilized by lidocaine-like drugs. In general, from bacteria to yeast and from worms to flies, anesthetics induce a broadly similar disruption of function [18]. Even in plant cells, it was found that common anesthetics induce a mitosis arrest, similar to cholchicine, which was considered as “narcotized cell division” [19]. The identification of GABA-regulated ion channels in plants suggests a direct action of anesthetics on plant metabolism with an impact on plant growth, development, and stress [20]. Likewise, the presence of a GABA/GABA receptor system in amoebae, such as *Dictyostelium discoideum* [21], makes it likely that this system is also present In *Physarum*.

Here, we present data showing the presence of transcripts in *Physarum* which share high similarity with the GABA_b_ receptor in humans and rats. Whether a putatively propofol-binding GABA_a_ homologue is also expressed in *Physarum* could not be said so far. However, the data presented here speak for a functional GABA/GABA receptor system in *Physarum*, which could have an influence on learning and memory by changing the migration behaviour. Evidence from *Dictyostelium discoideum* suggests an effect of GABA on spore encapsulation and metabolic profile [21]. Further studies must show whether there is a link with the observed “immobilising” effect. However, the exact physico-chemical nature of the memory-storing medium/process is still controversial, and it is questionable whether there is only one mechanism. It has been suggested that elements of the cytoskeleton, especially the microtubules, function as a storage medium for memory [22]. According to this model, the Ca^2+^-calmodulin kinase II (CaMKII) “prints” information via phosphorylation onto the microtubule lattice as ordered arrays of binary “bits”. Since the cytoplasm of *Physarum* contains a three-dimensional network of microtubules [23,24], this theory offers a mechanistic explanation for learning and memory even in single-celled organisms. Corroboratively, it has long been suspected that microtubules are a functional target for the action of anesthetics [18,25,26]. The results of our experiments support this assumption by showing both learning and propofol-induced amnesia in *Physarum*. However, our experiments using the 1-level Y-maze show that *Physarum* prefers the branch with MCT/LCT, and it is currently unknown whether *Physarum* metabolises it. Does *Physarum* not like the taste of propofol? From our human perspective, it is difficult to describe the behavior of an organism such as *Physarum* with the words “like” or “dislike”. Instead, one might rather assume that propofol acts as a repellent. It has been demonstrated that *Physarum*’s growth patterns can be controlled by culture conditions [27] and the addition of repellents such as quinine, caffeine, or sodium chloride [28,29]. Interestingly, if *Physarum* is repeatedly rewarded with food after crossing a barrier with sodium chloride, the slime mold habituates to the negative stimulus overcoming the initial aversion [11]. This was not observed in our experiments: *Physarum* does not habituate in the presence of propofol-soaked food.

The above concept of information processing by microtubules is undoubtedly speculative, and other possibilities for information storage and retrieval should be considered. Recent studies on *Physarum* show that environmental stimuli leave an imprint on the morphology of the network by modulating the hierarchy of tube diameters, which may serve as memory [30,31]. This supports the theoretical assumption of a self-model at the cellular level [32]. However, there are also indications of other mechanisms. For example, studies from the 1960s found evidence for RNA-based memory, at least in planarians [33,34]. We hope that this work will stimulate further studies deciphering the underlying mechanism.

## 4. Materials and Methods

### 4.1. Culturing of Physarum polycephalum

*Physarum polycephalum* (WT31, haploid wildtype), generously provided by Professor Dr. Wolfgang Marwan (Otto-von-Guericke-University, Magdeburg, Germany), was cultured as described [35]. Briefly, slime mold was propagated on solid agar plates supplemented with 5 g/L glucose, 5 g/L soytone (Difco-Bacto), 1 g/L KH_2_PO_4_, 0.45 g/L CaCl_2_, 1 × 10^−8^ M FeCl_3_, 0.17 mg/L ZnSO_4_, 1.77 g/L citric acid, 2.5 mg/L biotin, and 20 mg/L thiamine (Roth, Karlsruhe, Germany). The pH was adjusted to 4.6. After autoclaving, the molten agar was supplemented with 5 mg/L hematin (Sigma-Aldrich, Taufkirchen, Germany) before casting into plates. Spherules were generated by placing sterile strips of filter paper on growing cultures. Dried filter strips can be stored at −20 °C for at least one year. For revitalization, the filter strips were placed on agar plates and left for 8–14 days in the dark until the newly formed plasmodium was transferred to fresh medium plates.

### 4.2. Experimental Procedure

Proceeding from a rectangle-shaped chamber, a maze of five following Ys milled in acrylic glass was started. The maze was filled with 1.25% molten agar (Sigma) solution without nutritive additives. A sterilized oat flake was placed at the tip of the left arm of each Y except for the last one where no oat flake was placed. Each oat flake was soaked with 3 µL of the anesthetic compound Propofol-^®^Lipuro (1%) or the reference solvent consisting of 10% MCT/LCT emulsion, both generously supplied by Dr. Frank Nieber (B. Braun Melsungen AG, Melsungen, Germany). The decision for “right” or “left” was evaluated according to which of *Physarum*’s plasma strands reached the tip of one Y-branch first. If both tips were reached at the same time, this was considered as “equal”. In order to test the general preference, a depot of 3 µL of MCT/LCT and 3 µL of Propofol-^®^Lipuro without an oat flake was placed in the tips of a 1-level Y-maze.

**Figure 4:**
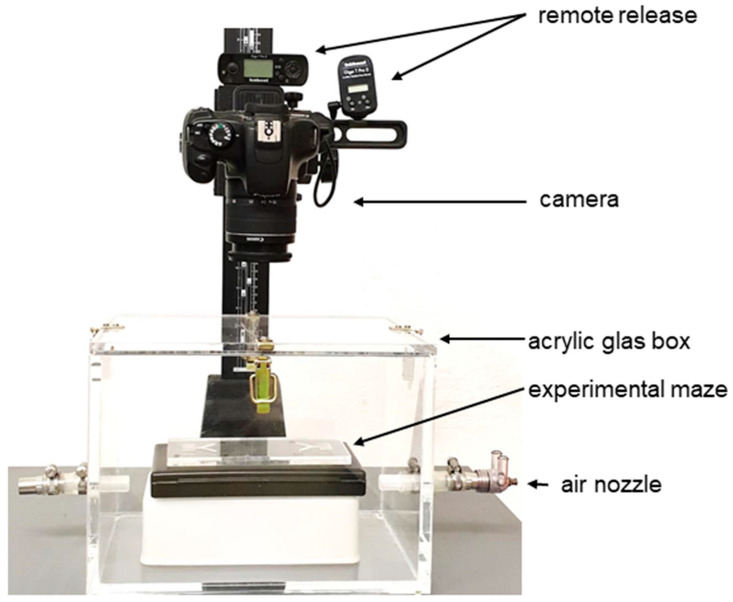
**Experimental set-up**. Acrylic glass container providing a humidified and semisterile atmosphere equipped with a heightened base carrying the experimental maze. Above this, attached to a stand controlled by an automated remote control, a camera took photographs.

Finally, the starting chamber was evenly inoculated with *Physarum polycephalum,* taken from medium plates (as described above). For each experiment, naïve *Physarum* samples were taken, which had not been tested before. Then, the maze was placed in a self-built acrylic glass container (Figure 4) to avoid contaminations.

The bottom of the chamber was layered with distilled water to maintain a humidified atmosphere. Two nozzles at each side allowed the exchange of air. A camera (Canon EOS 1100D) mounted on a stand connected to a remote controller captured photos every 15 min.

### 4.3. Statistics

Statistical evaluation was performed using a permutation test (MATLAB, The MathWorks, Natick, MA, USA). Each test was based on 10,000 permutations, with each permutation consisting of the same number of observations as the original data set. At each bifurcation, the possible outcomes were left and right. Cases where Physarum showed no distinct preference for one direction were omitted from the statistical analysis. Both directions were considered to be independent. We calculated the confidence intervals of the probabilities of turning left or right, assuming no difference between the two directions (H0). For each bifurcation, the distribution of probabilities to turn left or right was calculated. The dashed lines indicate the mean, and the dotted lines indicate the confidence intervals of two times the standard deviation. Values greater or less than this limit indicate statistical significance (*p* < 0.05).

## 5. Conclusions

Propofol is an intravenous anesthetic that has revolutionized systemic anesthesia. Although it has minimal effects on respiration and heart rate, there are adverse effects on the central nervous system, including disorientation and memory impairment. The data presented here from a single-celled organism confirm that memory is not necessarily dependent on the presence of a nervous system. Interestingly, in the presence of propofol, *Physarum*’s ability to find the rewarding oatmeal decreases. It is not yet clear whether this is due to a propofol-specific effect on memory formation or a repelling effect. Future studies will have to address this question.

## Figures and Tables

**Table 1:** Comparison of GABA receptor transcripts.

Query	UniProt	Name	Score	E-Value
PhyPoly_transcript_03383	sp|O88871	GABR2_RAT Gamma-aminobutyric acid type B receptor subunit 2	109	1 × 10^−22^
PhyPoly_transcript_03383	sp|Q80T41	GABR2_MOUSE Gamma-aminobutyric acid type B receptor subunit 2	108	3 × 10^−22^
PhyPoly_transcript_03383	sp|O75899	GABR2_HUMAN Gamma-aminobutyric acid type B receptor subunit 2	108	3 × 10^−22^
PhyPoly_transcript_04011	sp|Q9Z0U4	GABR1_RAT Gamma-aminobutyric acid type B receptor subunit 1	77	8 × 10^−13^
PhyPoly_transcript_04011	sp|Q9WV18	GABR1_MOUSE Gamma-aminobutyric acid type B receptor subunit 1	77	8 × 10^−13^
PhyPoly_transcript_04011	sp|Q9UBS5	GABR1_HUMAN Gamma-aminobutyric acid type B receptor subunit 1	77	8 × 10^−13^

## Data Availability

Not applicable.
